# Drug delivery systems based on polyethylene glycol hydrogels for enhanced bone regeneration

**DOI:** 10.3389/fbioe.2023.1117647

**Published:** 2023-01-30

**Authors:** Shouye Sun, Yutao Cui, Baoming Yuan, Minghan Dou, Gan Wang, Hang Xu, Jingwei Wang, Wen Yin, Dankai Wu, Chuangang Peng

**Affiliations:** Orthopaedic Medical Center, Second Hospital of Jilin University, Changchun, China

**Keywords:** PEG, drug delivery system, hydrogel, biomaterial, bone regeneration

## Abstract

Drug delivery systems composed of osteogenic substances and biological materials are of great significance in enhancing bone regeneration, and appropriate biological carriers are the cornerstone for their construction. Polyethylene glycol (PEG) is favored in bone tissue engineering due to its good biocompatibility and hydrophilicity. When combined with other substances, the physicochemical properties of PEG-based hydrogels fully meet the requirements of drug delivery carriers. Therefore, this paper reviews the application of PEG-based hydrogels in the treatment of bone defects. The advantages and disadvantages of PEG as a carrier are analyzed, and various modification methods of PEG hydrogels are summarized. On this basis, the application of PEG-based hydrogel drug delivery systems in promoting bone regeneration in recent years is summarized. Finally, the shortcomings and future developments of PEG-based hydrogel drug delivery systems are discussed. This review provides a theoretical basis and fabrication strategy for the application of PEG-based composite drug delivery systems in local bone defects.

## 1 Introduction

Bone defects occur for many reasons, such as severe trauma, tumors, and systemic diseases (Qiao et al., 2020). Most bone defects spontaneously repair, with few (5%–10%) requiring surgical treatment for recovery (Zhong et al., 2018). At present, autologous bone transplantation is the preferred clinical treatment for such bone defects. Although autografts promote bone repair, their main disadvantages are a limited bone mass, donor site pain, and infection (Gong et al., 2018; Boteanu et al., 2020). Allogeneic bone is rich in source, not limited by shape or size, and has good biological activity, showing good bone tissue repair (Bai et al., 2017). However, allograft bone transplantation still has a high failure rate, which mainly manifests as delayed healing, non-union and fatigue fracture of the bone graft, immune response, and bone resorption (Balagangadharan et al., 2018). Following the principle of tension stress, bone transport technology using active bone graft to treat large segmental bone defect has become an important surgical treatment. However, its main disadvantages are the long placement time of external fixator and the risk of bone non-growth (Catagni et al., 2019; Dahl and Morrison, 2021). Therefore, it is necessary to develop alternative grafting materials.

Bone tissue engineering (BTE) is a cross-discipline field that combines the technology and knowledge of materials engineering and biological factors to enhance bone tissue regeneration (Cui et al., 2019). It includes treatment based on genes, cells, and cytokines, and has been reported to be a promising strategy to replace autologous bone transplantation and allogeneic bone transplantation (Liu C. et al., 2021; Gaihre et al., 2021). The latest developments in BTE have identified and promoted biomaterials such as hydrogels as a meaningful way to enhance bone regeneration (Xie et al., 2021). Due to their excellent biocompatibility, controllable biodegradability, and injectable methods, hydrogels have been recognized as an important research topic in the field of medical repair and regeneration (Kim et al., 2020). Hydrogels are water-swellable polymer materials that can form a 3D network *via* cross-linking reactions between hydrophilic polymers (Zhao et al., 2022). Due to their bionic characteristics and certain mechanical strength, hydrogels play a role in connecting and supporting cells and tissues during bone repair, and also determine certain physiological activities such as cell survival, growth, proliferation, and differentiation, providing a certain microenvironment or extracellular matrix (ECM) for stem cells (Zhang et al., 2021b). Therefore, they are often used as carrier materials in bone defect repairs and are an important focus of research in tissue engineering. A variety of osteogenic substances, such as bone morphogenetic protein (BMP) and vascular endothelial growth factor (VEGF), can be loaded into the hydrogel matrix network to form a drug delivery system (Ren et al., 2017; Bal et al., 2021).

Compared with other polymers, PEG is an important hydrophilic polymer. Due to its non-toxicity, non-immunogenicity, good biocompatibility, and anti-protein adsorption, it has been widely used in the biomedical field for drug delivery, tissue engineering, and surface modification (Liu et al., 2019; Gan et al., 2021). Moreover, the hydrophilicity of PEG has been demonstrated to be adjustable, increasing with the increase of molecular weight and decreasing with the increase of temperature (Ganapathi et al., 2015). As a carrier material, PEG has an active hydroxyl end, so it can be combined with a variety of drug active molecules to form a drug delivery system. Among them, PEG with two hydroxyl end groups is most commonly used because it can also be modified by other functional groups for further applications, such as forming functional hydrogels with other monomer macromolecules or particles by photopolymerization (Wang D. et al., 2018). The swelling ratio and mechanical strength of PEG-based hydrogels can be controlled by changing the molecular weight and specific gravity of PEG. In addition, some PEG derivatives have pH sensitivity, and they can form pH-intelligent responsive hydrogels on bone defects in acidic microenvironments caused by trauma to obtain a drug release pattern more in line with the characteristics of the local microenvironment, thereby promoting bone regeneration (Kono, 2014; Ghorpade et al., 2018). PEG-based hydrogels are formed by integrating PEG with other substances through different methods such as free radical polymerization and functional group reaction (Day et al., 2018; Jiang et al., 2020) PEG-based hydrogels showed unique advantages due to numerous functions (Whitehead et al., 2018; Filova et al., 2020). Drug delivery systems based on PEG hydrogel have broad application prospects and can be applied to biomedical and pharmaceutical materials.

**SCHEME 1:**
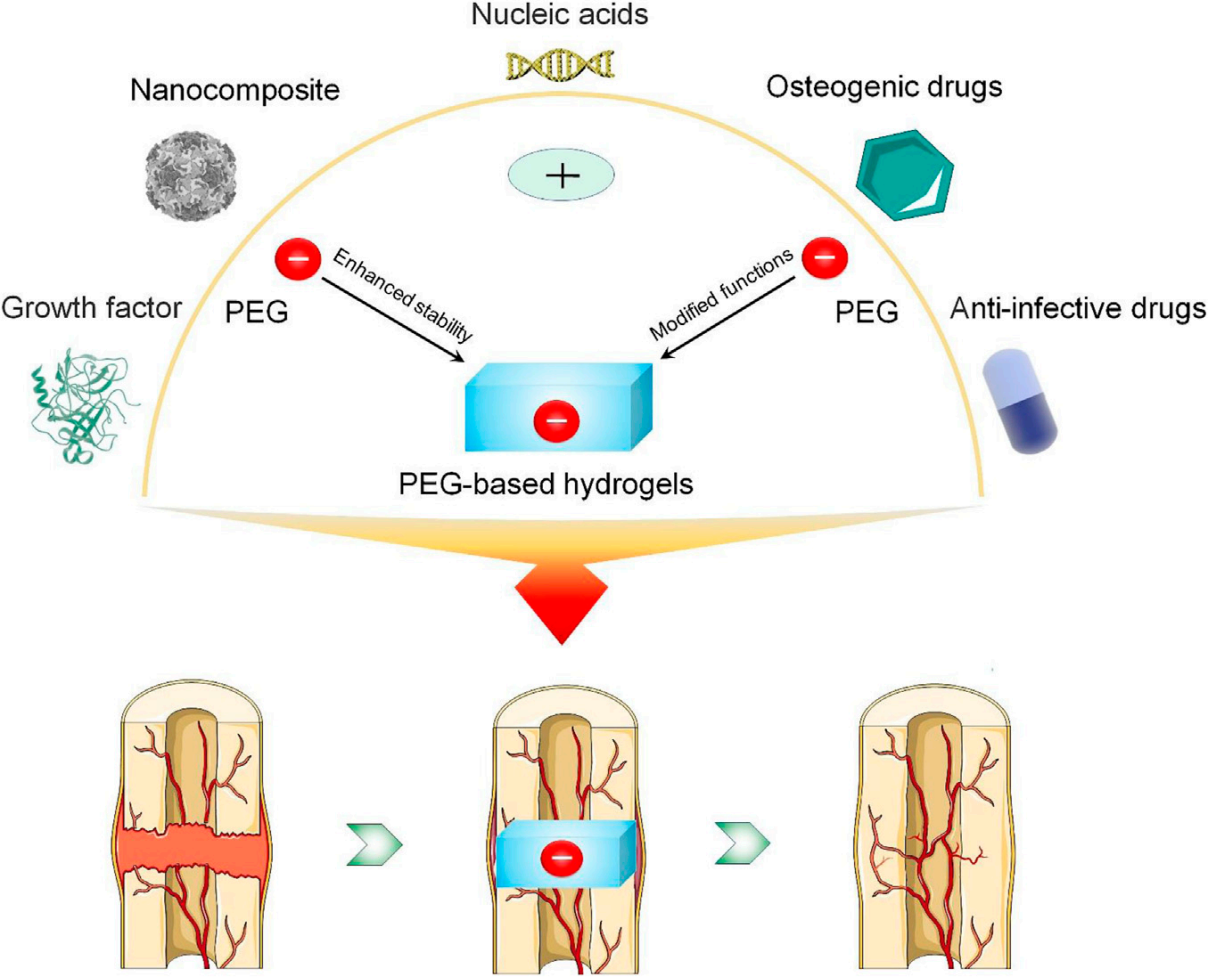
Modification of PEG-based hydrogels and construction of a drug delivery system to enhance bone regeneration.

Over the past decade, the application of PEG-based hydrogels in various fields has become an important research topic. Although PEG hydrogels have not been clinically applied to treating bone defects on a large scale, they have unique properties and can provide structural support for the defect site and allow bone defects to be repaired through the internal healing mechanism (Wang et al., 2019). However, the current understanding and application of PEG hydrogel drug delivery systems in bone regeneration are not sufficiently comprehensive. This review begins with the structure of PEG, focusing on a variety of methods to improve the stability of PEG-based hydrogel drug delivery systems and synthesize PEG-based hydrogels with antibacterial and osteogenic properties. On this basis, the application of PEG-based hydrogel drug delivery systems in enhancing bone regeneration in recent years is summarized. Finally, the development prospects of PEG-based hydrogel drug delivery systems are discussed (Scheme 1). This review will provide a theoretical basis and advanced strategies for PEG-based biomaterials to treat bone defects.

## 2 Fabrication strategy of PEG-based hydrogels

Local drug delivery systems composed of osteogenic active substances and biomaterials provide a new strategy for bone regeneration (Adjei et al., 2019). An ideal drug delivery system provides a sustained release of a drug in a controlled manner to maintain an appropriate drug concentration in the defect area without affecting healthy bone tissue (Cheng et al., 2018). The carrier material plays a vital role in the release curve (Kondiah et al., 2020). The application range of PEG molecular weight is very wide. Due to its biological inertia and good biocompatibility, oligonucleotides, proteins, peptides, and drugs can be modified by the PEG covalent bond, which can effectively prolong the time *in vivo* and reduce toxic and side effects (Sung et al., 2020). At the same time, PEG is soluble in both water and most organic solvents, and can be excreted through the kidney without accumulation *in vivo*. It is a widely used biological material and medical excipient recognized by the US Food and Drug Administration (FDA) (Frassica et al., 2019). Therefore, PEG is a suitable option for constructing carriers. The functional modification of PEG can imbue it with excellent drug release effects. In addition, PEG can also be directly and biologically modified to form bioactive hydrogels with antibacterial or bone-promoting functions (Table 1) (Tripodo et al., 2018; Tikhonov et al., 2020). In this section, we will outline the effects of PEG with different structures on drug release, as well as measures to improve the stability of PEG crosslinked hydrogels *in vivo*. Finally, we will introduce the biological modification of PEG to enable its corresponding functions.

**TABLE 1:** Summary of the functional and biological modified PEG.

Materials	Compound method	Characters
PEG/PLA	Using lactide as raw material, four-arm PEG as tetrahydroxy initiator and Sn(Oct)_2_ as catalyst	Good mechanical strength, excellent stability, injectability
PEG/PLGA	Using L-lactide and glycolide as raw material, PEG as tetrahydroxy initiator and Sn(Oct)_2_ as catalyst	Accurate temperature sensitivity, appropriate degradability
PEG/Alginate	Using PEG-NH_2_, oxidized alginate, gelatin as raw materials, *via* Schiff base reaction	Osteogenic function, good biocompatibility, injectability
PEG/Nanoclay	Using PEG diacrylates and nanoclay as raw material, in an ultraviolet light crosslinker	Osteogenic function, good mechanical properties, excellent stability
PEG/CS	Using mPEG-acrylate and CS as raw material, *via* graft copolymerization	Antibacterial function
PEG/Ag^+^	Using four-arm-PEG-SH and Ag^+^ as raw material, *via* dynamic binding of -SH to Ag^+^	Antibacterial function, excellent self-healing, injectability
PEG/Hyaluronic acid	Using PEG, poly (N-(2-hydroxypropyl) methacrylamide lactate), hyaluronic acid as raw material, *via* Michael addition reaction	Osteogenic function, good stability, temperature sensitivity

### 2.1 Structure of PEG

The structural formula of PEG is HOCH_2_ [CH_2_OCH_2_]nCH_2_OH or H [OCH_2_CH_2_] nOH, which is the general term for ethylene glycol polymers with an average molecular weight of 200–8,000 or more (Tohamy et al., 2021). With various initiators and synthesis methods, the structure of PEG is also variable, although the most common structures are linear, four-arm, six-arm and eight-arm (Table 2) (Dirisala et al., 2020; Zhao et al., 2020; Song et al., 2021). Different arms of PEG can be modified by different groups such as amino, maleic amide, and carboxyl moieties, which provide sites for its combination with other materials and drugs (Zhang D. et al., 2021; Park et al., 2022). This is a unique property of PEG materials. Linear PEG with heterobifunctional groups can be directly attached to biomolecules to form a drug delivery system (Liu et al., 2018). However, linear PEG has certain limitations, such as low drug loading efficiency. In addition, micelles formed by linear PEG and other polymers easily collapse and lose their pharmacological effects; thus, it is difficult to meet the time required for bone defect repair (Ma et al., 2022). A four-arm PEG increases the number of modified end groups, thereby increasing the drug loading and prolonging the degradation time *in vivo*. Four-arm PEG hydrogels can be synthesized *via* a simple A-B reaction based on Flory’s classical theory by combining symmetrical tetrahedral macromonomers, and they exhibit the same elastic modulus as articular cartilage (Apostolides et al., 2018).

**TABLE 2:** Summary of the structure and characteristics of PEG.

Types	Structures	Advantages	Disadvantages
Linear PEG	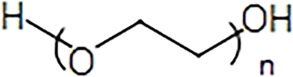	Simple synthesis process, Excellent water solubility	Low drug loading
Four-arm PEG	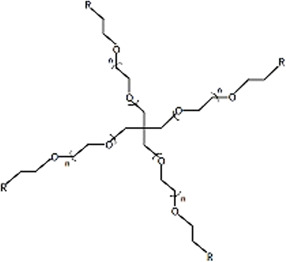	High drug loading, Multiple functional selectivities, Improve the solubility of drugs, Low critical micelle concentration	Easy internal crosslinking, Slow gelation speed
Six-arm PEG	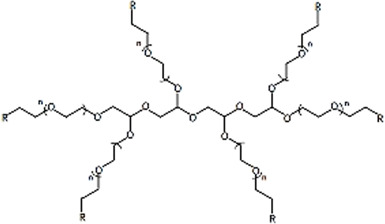		
Eight-arm PEG	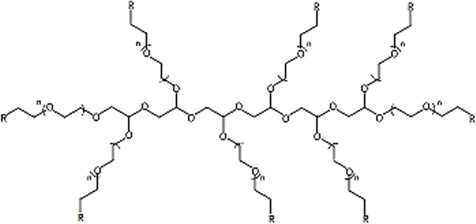		

Eight-arm PEG has more modifiable groups, which can improve functional selectivity and drug loading (Xue et al., 2021). For instance, it can be combined with two drugs at the same time through different groups to improve the functionality of the drug delivery system. Compared with linear and four-arm PEG, eight-arm PEG hydrogels have lower mechanical properties (Ma et al., 2016). Since intramolecular aggregation prevents cross-linking of the chain, the eight-arm PEG has a tighter structure, resulting in a smaller binding capacity. The many structures of the eight-arm PEG will inevitably lead to a prolonged gel-forming time (Zhang et al., 2020).

Although most of the biological properties of PEG are very satisfactory, some PEG crosslinked hydrogels also have certain shortcomings, such as rapid degradation under aerobic conditions and the inability to exist in the required site as a continuous carrier (Herten et al., 2009; Pohlit et al., 2015). Therefore, improving and modifying the PEG hydrogel to a certain extent can promote its application prospects.

### 2.2 Enhanced stability

The stability *in vivo* is one of the key factors that determine whether the drug delivery system can effectively play a role. An appropriate degradation rate is accompanied by an ideal drug release curve. PEG is highly unstable because of its ether structure, which can form peroxides (Thoma et al., 2017). In view of this shortcoming, PEG can be block copolymerized with other polymers or grafted to slow down the degradation rate (Chen et al., 2018; Fer et al., 2021). Polylactic acid (PLA) is a biodegradable material with good solvent resistance and thermal stability (Vasilyev et al., 2021). Combination with PLA can effectively improve the stability of PEG. Using lactide as the raw material, four-arm PEG as the tetrahydroxy initiator and Sn(Oct)_2_ as the catalyst, Jun et al. (2008) prepared a PEG-PLA diblock copolymer and its stereocomplex hydrogel with tailorable structure and chain length. An *in vitro* degradation experiment confirmed that the 22 wt% PEG-PLA hydrogel degraded slowly, which was attributed to the wide range of stereocomplex regions of the block copolymer. However, the hydrogels synthesized in this way have a high swelling rate, and the swollen hydrogels have poor strength. The number of PLA repeat sequences plays a decisive role in the degradation time and drug release rate of the hydrogel. Increasing the number of PLA repeats can accelerate the degradation rate of the PEG/PLA composite gel, but the pore size of the hydrogel will increase, and the control of drug release is more accurate. Based on PEG-PLA, Wang Y. et al. (2018) synthesized poly (ethylene glycol)-poly (lactic acid)-dimethacrylate (PEG-PLA-DM) by microwave-assisted methacrylic acidization of the diblock copolymer. Studies have shown that when the PLA repeat number is five, the hydrogel system shows better drug release control.

**FIGURE 1:**
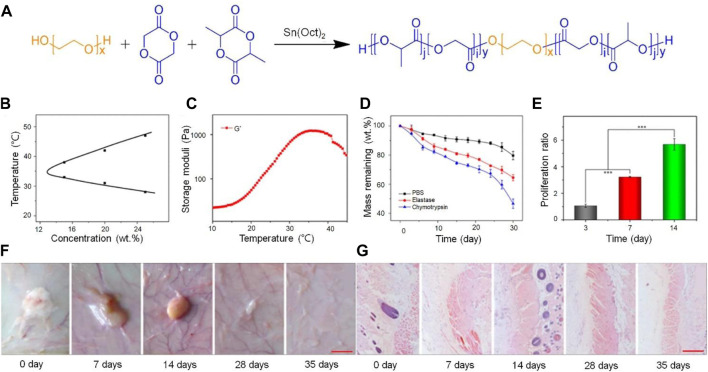
Combining PLGA with PEG, which improved the stability of the drug sustained release system (Li et al., 2016). **(A)** Schematic illustrations of synthesis pathways of PLGA-PEG-PLGA triblock copolymer. **(B)** Temperature-dependent sol−gel phase transitions of the copolymer solutions in PBS at different concentrations. **(C)** G′ of the copolymer in PBS at a concentration of 20 wt%. **(D)** *In vitro* degradation of the thermogel in PBS. **(E)** Proliferation behavior of BMSCs encapsulated in the thermogel. (***p < 0.001). **(F)** *In vivo* degradation of the thermogel in rats, and **(G)** H&E staining of the skin. The first red scale bar represents 5 mm. The second red bar represents 200 µm. PLGA, Poly (L-lactide-co-glycolide); H&E, hematoxylin and eosin; PBS, phosphate buffered saline; BMSCs, bone mesenchymal stem cells. Copyright ^©^ 2018, Mary Ann Liebert, Inc. Reproduced with permission from Mary Ann Liebert, Inc.

Poly (L-lactide-co-glycolide) (PLGA) is a biomaterial with good cytocompatibility approved by the FDA (Javier Lopez-Cano et al., 2021). Introducing PLGA into a PEG terminal group is an effective method to enhance the stability of PEG. The combination of PLGA and PEG not only improves the biocompatibility of the drug delivery system, but also enhances its stability. PLGA-PEG-PLGA was synthesized by ring-opening polymerization with L-lactide and glycolide as the raw materials, Sn(Oct)_2_ as the catalyst, and PEG as the macromolecular initiator. As the temperature increases, the hydrophobic PLGA block in PLGA-PEG-PLGA will participate in different micelles, resulting in extensive aggregation. A sudden increase in the number of aggregated micelles will lead to a sol-gel transition. The thermosensitive hydrogel PLGA-PEG-PLGA was injected subcutaneously into mice in a minimally invasive manner, and the hydrogel was completely degraded after 5 weeks. *In vitro* degradation experiments showed that about 30% of the 20 wt% PLGA-PEG-PLGA hydrogel did not degrade after 30 days, thus providing suitable conditions for BMP-2 release and cell growth (Li et al., 2016) (Figure 1).

In addition, the combination of PEG and natural hydrogels can also effectively improve stability. Alginate has been widely used in the biomedical field due to its stability, high viscosity, and gelling properties in aqueous solutions (Kamaraj et al., 2021). The cross-linking degree of a self-crosslinking injectable hydrogel prepared by the reaction of PEG, oxidized alginate, and gelatin through a Schiff base reached 71.73% ± 0.22% (Naghizadeh et al., 2018); its stability was also significantly improved, and the *in vitro* degradation time can reach 30 days. Compared to chemical crosslinking agents, the Schiff base reaction is non-cytotoxic and does not require additional crosslinking agents such as initiators or light sources. Interestingly, the addition of nanomaterials to PEG crosslinked hydrogels can also delay the degradation rate and increase stability. Laponite is the most commonly used nanoclay, which is composed of SiO_2_, MgO, Li_2_O, Na_2_O (Podaru et al., 2022). Zhai et al. (2018) obtained a PEG diacrylates-clay nanocomposite hydrogel by modifying the hydroxyl group of PEG and crosslinking with nanoclay. The physical dispersion between nanoclay particles and PEG chains and the chemical crosslinking in PEG network make the nanocomposite hydrogels have higher stability and mechanical properties than pure PEG hydrogels. With the increase of nanoclay concentration, the cross-linking is more stable. The drug delivery system had high stability, where the release rate showed an upward trend in the first 7 days, and then slowed down. At 21 days, 165–295 μg/mL of Si^4+^ and 75–125 μg/mL of Mg^2+^ could still be detected, and both concentrations could effectively induce bone regeneration.

### 2.3 PEG-based hydrogels with antibacterial function

In addition to the modification of physical and chemical properties, biological modification has also been a hot topic in recent years (Yang et al., 2021). Antibacterial properties are an important part of PEG hydrogel modification, which enable its good application prospect in infectious bone defects (Wei et al., 2018). PEG-based materials with antibacterial modification will provide an ideal drug carrier for local treatment of infectious bone defects.

Chitosan (CS) is used for the synthesis of hydrogels due to its biocompatibility, biodegradability, and abundant natural resources. At the same time, CS has positively charged quaternary ammonium groups in its structure, so it can inhibit the growth and proliferation of bacteria through electrostatic interaction (Kowalczyk et al., 2021). In one study, a CS/PEG hydrogel was synthesized by graft copolymerization. The composite hydrogel system could maintain the continuous delivery of CS, and, with the increase of CS concentration, the antibacterial effect became more obvious (Peng et al., 2020).

However, the low solubility of CS under physiological and neutral conditions limits its application. Glycol chitosan (GCS) is a water-soluble CS derivative that can increase the solubility of chitosan in physiological and neutral conditions, and has the biological characteristics applied in BTE (Sautrot-Ba et al., 2019). GCS-PEG crosslinked hydrogels exhibit significant antibacterial activity. Tripodo et al. (2018) prepared GCS-PEG hydrogel with diepoxy PEG (PEGDE) as crosslinking agent at 37°C. The use of PEGDE can avoid purification after production. At the same time, the concentration of PEGDE can change the crosslinking degree of hydrogels, thus affecting the antibacterial ability. Studies confirmed that the inhibition rate of the hydrogel group on *Staphylococcus aureus* (*S. aureus*) was close to 90%. *S. aureus* is the most common bacteria that causes infectious bone defects (Zhang S. et al., 2016). Ag^+^ can react with bacteria and inhibit the synthesis of proteins to either cause the destruction of the inherent components of bacteria or produce functional disorders, leading to the death of bacteria and the inability to grow and reproduce (Yuan et al., 2020). Ag^+^ was crosslinked with modified four-arm-polyethylene glycol-thiol (four-arm-PEG-SH) by Ag-S coordination to form a dynamic coordination injectable hydrogel. The study showed that the hydrogel group had a wide antibacterial spectrum and a good inhibition effect on *S. aureus* and *Escherichia coli* (*E. coli*) (Cheng et al., 2020).

### 2.4 PEG-based hydrogels with osteogenic function

PEG-based hydrogels exhibit extraordinary osteogenic induction ability when mixed with bone-promoting materials (Liu W. et al., 2021). As an inorganic component of normal bone tissue, hydroxyapatite (HA) has outstanding compatibility and bone conductivity (Li et al., 2019). Adding HA directly into a PEG-based hydrogel endows the composite hydrogel with osteogenic function. Nejadnik et al. (2012) confirmed that the addition of HA nanoparticles significantly increased the mineralization ability of PEG-based hydrogels. Similarly, calcium phosphate nanoparticles composed of calcium ions and phosphate ions in different proportions can also enable osteogenic function in PEG crosslinked hydrogels. The injectable hydrogel was synthesized by the reaction of four-arm PEG-SH with calcium phosphate nanoparticles. The composite hydrogel enhanced the ECM mineralization ability and alkaline phosphatase activity of mouse embryo osteoblast precursor cells, and significantly promoted the healing of critical skull defects in rats (Cheng et al., 2020).

**FIGURE 2:**
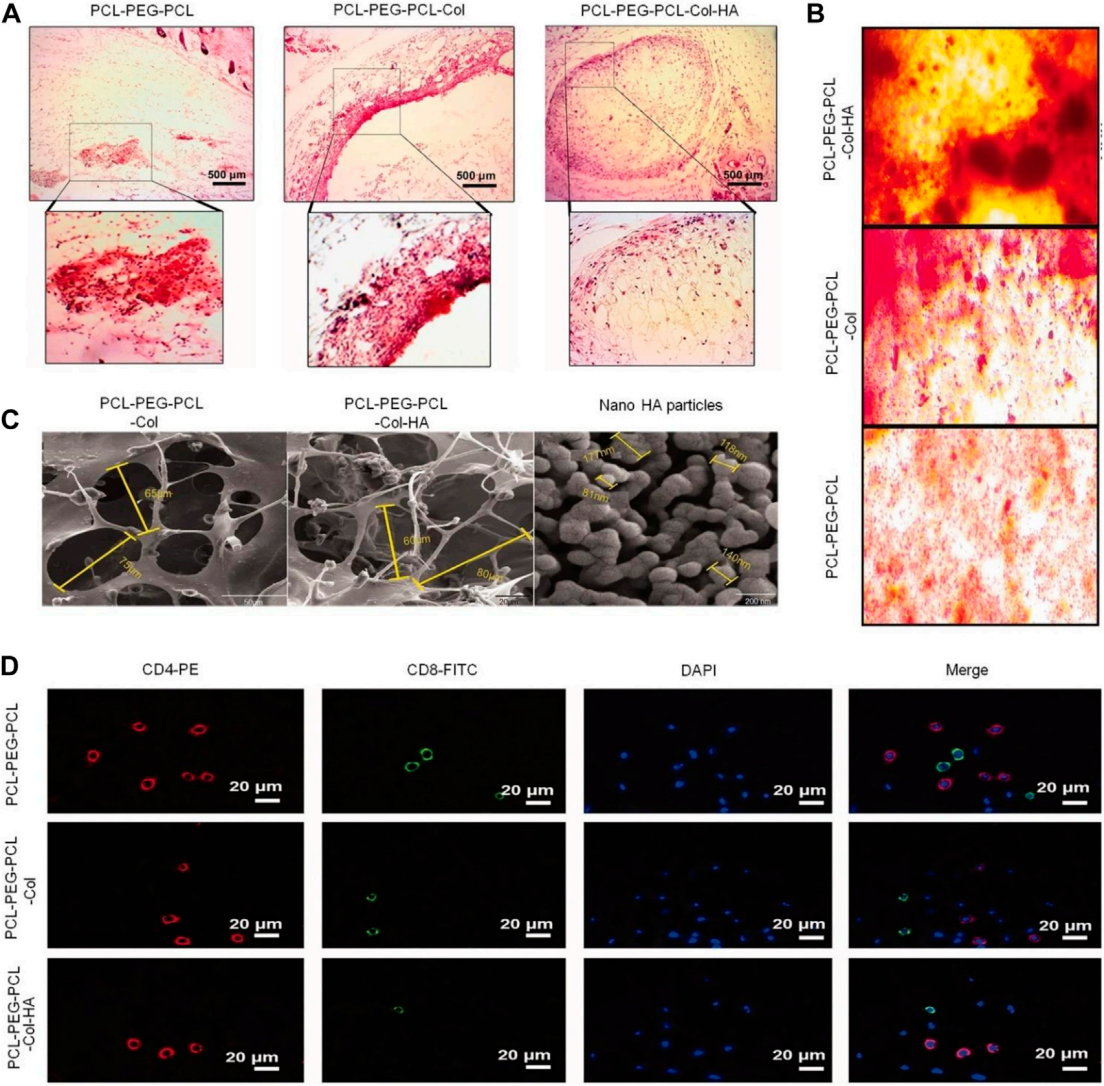
The addition of collagen imbued PEG-based hydrogels with osteogenic function (Hassanzadeh et al., 2021). **(A)** H&E staining performed at the injection site after the hydrogel was injected subcutaneously into rats for 14 days. **(B)** Alizarin red staining of BMSCs seeded on hydrogels after 14 days. **(C)** FE-SEM image of synthesized nanoparticles, scaffolds, and corresponding diameter distributions. **(D)** Blood CD8/CD4 lymphocyte ratio using IF staining. H&E, hematoxylin and eosin; BMSCs, bone mesenchymal stem cells; FE-SEM, field-emission scanning electron microscopy; IF, immunofluorescence. Copyright ^©^ 2021 The Author(s). Reproduced with permission from Taylor and Francis Group.

The connection between bone and cartilage is very important for the repair of bone tissue. Hyaluronic acid, a degradable component of the ECM of connective tissue, has been used by many studies as a good 3D biomaterial for cartilage and BTE (Yuan et al., 2019). Agas et al. (2019) crosslinked thioated hyaluronic acid with the PEG-based thermosensitive hydrogel modified by the vinyl sulfone group through the Michael addition reaction. The mechanism of simultaneous physical (thermal) and chemical (Michael reaction) crosslinking is to avoid premature dissolution of the polymer chain prior to chemical crosslinking. The hydrogel was gelled immediately after injection *in vivo*, ensuring the stability of the hydrogel *in situ* during the subsequent Michael addition crosslinking process. After local administration *in vivo*, the hydrogel exhibited the ability to restore bone mineralization and promote proteoglycan production. Similarly, as a kind of polymer collagen extracted from ECM, the hydrogel had the function of supporting cell adhesion, proliferation, migration, and differentiation. At the same time, the mineralization of collagen fibers plays a vital role in ensuring the structure and mechanical function of bone tissue (Hassanzadeh et al., 2021). The addition of collagen gives PEG-based hydrogels osteogenic function (Figure 2). Fu et al. (2012) successfully synthesized a new type of temperature-sensitive hydrogel composed of collagen and PEG. In a rabbit bone defect model, after the composite hydrogel was implanted for 4 weeks, micro-CT images and tissue sections showed that the hydrogel group had better bone regeneration capacity compared with the control group.

**FIGURE 3:**
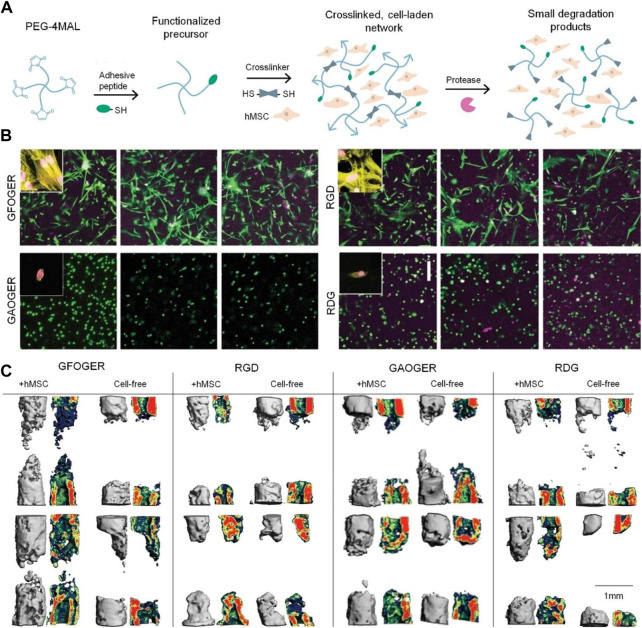
Integrin-specific hydrogel with osteogenic effect obtained by coupling GFOGER with a four-arm PEG monomer (Clark et al., 2020). **(A)** Four-arm PEG-maleimide hydrogel reaction scheme. **(B)** Hydrogel-encapsulated hMSCs cultured for 7 days and stained with Calcein-AM (green) and ethidium homodimer (magenta), scale bar = 200 µm. Insets show actin (yellow) and nuclei (magenta) stains. **(C)** Bone formation monitored with live animal µCT. Representative 3D reconstructions with sagittal mineral density heat maps. Scale bar = 1 mm μCT, micro-computed tomography; hMSCs, bone mesenchymal stem cells; 3D, three-dimensional. Copyright ^©^ 2020, The Author(s). Reproduced with permission from Nature Communications.

In addition to collagen, collagen mimic peptides (CMPs) have received extensive attention in recent years. Compared with collagen, CMPs have a higher stability and more intensive binding sites of cell surface receptors (Mhanna et al., 2014). Therefore, the application of CMPs to modify PEG has good prospects. GFOGER is a commonly used CMPs that can form a stable connection with PEG through terminal groups. GFOGER was coupled to the four-arm PEG of the maleimide group *via* a thiol group to obtain an integrin-specific hydrogel. The hydrogel showed a regulatory effect on human mesenchymal stem cells (hMSCs) adhesion and osteoblast differentiation (Clark et al., 2020) (Figure 3). Grafting GFOGER onto the PEG-based hydrogel not only allowed the composite gel to have osteogenic capability, but it also achieved a more significant osteogenic effect by loading substances such as BMP-2 and VEGF (Garcia et al., 2016). In addition to directly promoting osteogenic differentiation, GFOGER can have a synergistic effect with BMP-2. The dual drug-loading system of GFOGER and BMP2 has a more obvious promoting effect on bone regeneration than the single BMP2 system. In one study, low-dose BMP-2 was loaded into a GFOGER functional hydrogel and implanted into the bone defect of a mouse forelimb (Shekaran et al., 2014). Eight weeks after surgery, micro-CT showed that GFOGER could endow the PEG hydrogel with osteogenic ability, and low-dose BMP-2 further enhanced the bone repair characteristics of the PEG-based hydrogel.

## 3 Application of drug delivery system based on PEG hydrogels in BTE

PEG-based hydrogels materials can be loaded with drugs in their matrix, directly injected, or used as coating materials for bone defects (Cohen et al., 2022). Drug loading in the matrix can achieve a high drug loading rate and drug utilization rate. A large number of studies have confirmed that PEG-based hydrogels show an ideal drug delivery profile in bone defects (Yuan et al., 2019).

In this section, we will introduce the application of PEG-based hydrogels carrying an osteogenic growth factor, metal particles, nucleic acids, or drugs in enhancing bone defect healing. In addition, we also will introduce the application of PEG-based hydrogels loaded with anti-infective drugs in the regulation of the microenvironment of infectious bone defects.

### 3.1 PEG-based hydrogels loaded with growth factor

BMP is a group of highly conservative functional proteins with similar structures, belonging to the transforming growth factor-β superfamily (Aghali and Arman, 2020). BMP promotes bone regeneration mainly by its receptor binding to the ligand, phosphorylating the downstream Smad1/5/8, and then binding to Smad4 to enter the nucleus to activate the transcription factor (Miao et al., 2022). In addition to activating Smad-related classical signaling pathways, BMP can also activate Smad-independent non-classical signaling pathways, containing MAPKs, JNK, PI3K, and Akt (Yan et al., 2022; Zhou et al., 2022). At present, BMP is widely used in the field of orthopedics (Rojasawasthien et al., 2022). However, the side effects caused by the application of supraphysiological doses and its high cost have greatly limited the clinical application of BMP-2 (Santovena et al., 2017). Therefore, the design and production of an appropriate BMP-2 local release system is the key to improve the therapeutic effect and reduce the dosage and side effects.

PEG-based hydrogels, as an ideal delivery system of BMP-2, can maintain a local high concentration of BMP-2 for a period of time and effectively induce bone regeneration. Schoonraad et al. (2021) combined BMP-2 with PEG hydrogel chemistry by thiol-norbornene click chemistry, where covalent binding could provide a mechanism for dose control and location to improve the effectiveness and safety of using BMP-2. This sustained-release system significantly prolonged the release time of BMP-2 and promoted the osteogenic expression of mouse embryo osteoblast precursor cells through a Smad1/5/8 signaling pathway. Furthermore, PEG-based hydrogels can also be used as prominent carriers for stem cell transplantation. By locally loading BMP and stem cells, the hydrogels can also play a good role in promoting bone formation and reduce the dosage of BMP (Kong et al., 2020). Two eight-arm PEG precursor molecules were combined with lysine or glutamine receptors to form PEG-based biomimetic hydrogels with enzymatic crosslinking and high controllability. Skeletal stem cells (SSCs) and low-dose BMP-2 were directly encapsulated in PEG-based hydrogel to construct a drug delivery system to treat 4-mm skull defects in mice. Micro-CT and histological section staining demonstrated that a sufficient number of SSCs and a low dose of BMP-2 were effective in enhancing bone regeneration (Papageorgiou et al., 2019).

It is undesirable to excessively prolong the degradation time of PEG-based hydrogels *in vivo*. Because hydrogels rarely promote the inward growth of cells, it is easy to hinder the inward growth of tissues if they are not cleared for a long time. Moreover, the amount of BMP released will decrease with prolonged degradation time, which is insufficient to promote osteogenesis (Wang L. et al., 2020). Kossover et al. (2020) designed and synthesized a cell-free, biodegradable hydrogel composed of denatured albumin and PEG. BMP was loaded into a composite hydrogel by physical blending. Rat tibial defect experiments proved that some of the hydrogel was still undegraded at 13 weeks and had been wrapped by granulation tissue. This is disadvantageous for the continued ingrowth of bone tissue (Li et al., 2021). Therefore, the matching of the *in vivo* degradation time and bone regeneration rate should be considered in the design of hydrogels.

**FIGURE 4:**
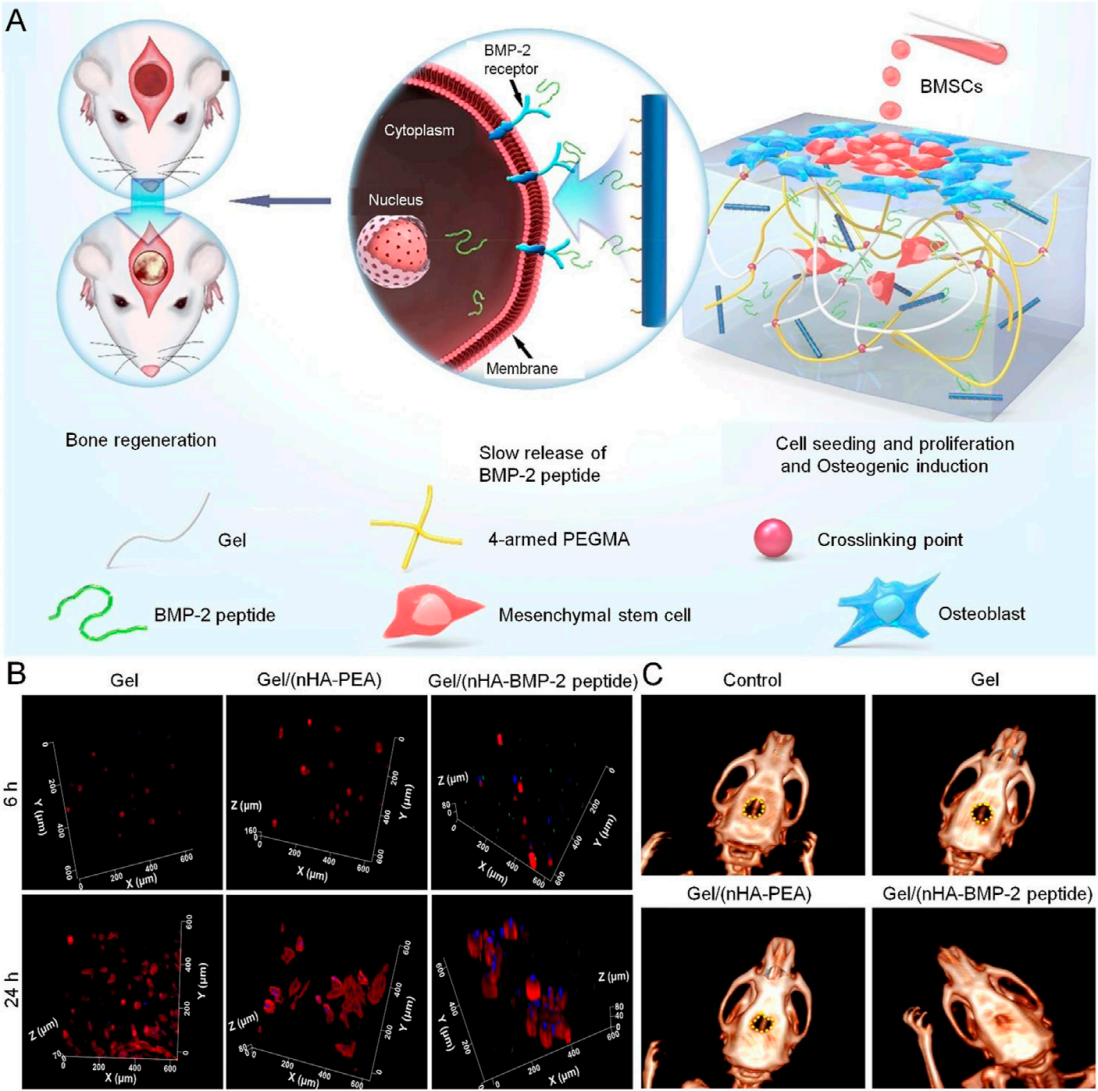
PEG-based hydrogel loaded with BMP-2 peptides for treatment of bone defect (Shao et al., 2018). **(A)** *In vitro* and *in vivo* potential of the composite for effective bone regeneration by slowing the release of the BMP-2 peptides. **(B)** BMSCs cultured within the hydrogels (3D culture mode) for 6 and 24 h **(C)** CT images of the rat calvarial defect model at 12 weeks. BMP-2, bone morphogenetic protein-2; BMSCs, bone mesenchymal stem cells; 3D, three-dimensional. Copyright ^©^ 2018, American Chemical Society. Reproduced with permission from American Chemical Society.

In recent years, BMP-2 derived peptides have provided an alternative solution to the BMP-2 growth factor. BMP-2 peptides have more stable structural characteristics and retains its osteogenic properties (Chao et al., 2021). PEG-based hydrogels carrying BMP-2 peptides have broad application prospects. To enhance bone regeneration more ideally, Shao et al. (2018) synthesized gel methacrylamide, four-arm PEG methacrylamide/inorganic HA-BMP-2 peptides complexes. BMP-2 peptides are covalently fixed with nano-HA to maintain biological activity and slow release. The BMP-2 peptides were connected with the gel by hydrogen bonding, which enhanced the biocompatibility between organic and inorganic components, and also increased the mechanical strength of the composite hydrogel. *In vivo* experiments showed that the composite hydrogel promoted bone regeneration after 12 weeks of implantation (Figure 4).

In addition, PEG-based hydrogels can also load a variety of growth factors to obtain different functions. As osteogenesis and angiogenesis are coupled and interact with each other in the repair process of bone defects, a drug delivery system with dual functions of promoting angiogenesis and osteogenesis will have good application prospects (Pekozer et al., 2021). VEGF and BMP-2 co-loaded into PEG-based hydrogel matrix can endow the gel with angiogenesis and osteogenesis functions. VEGF plays an important role in all stages of bone repair, including inflammation, callus formation, and callus remodeling. VEGF mainly acts on adjacent endothelial cells in a paracrine manner by binding to vascular endothelial growth factor receptors, regulating endothelial cell migration, proliferation, and vascular permeability (Kim et al., 2021). In addition, VEGF has a synergistic effect on BMP promoting bone regeneration (Geng et al., 2021). Egri and Eczacioglu (2017) loaded BMP-2 and VEGF into lyophilized PLA-PEG-PLA hydrogel to achieve a sequential release of the two growth factors. BMP-2 was loaded into the hydrogel matrix by BMP-2 dimethyl sulfoxide copolymer solution. VEGF was loaded into the hydrogel matrix by mixing 1 ml 1% gelatin solution in water/ethanol (50:50). The drug release curve showed that the release rate of VEGF was faster in the first 7 days, and the release rate of VEGF was 60% on the seventh day, whereas the release of BMP-2 was long-term and slow, which met the needs of the body in the time sequence of the two factors during bone regeneration.

### 3.2 Osteogenic nanoparticle-integrated PEG-based hydrogels

**FIGURE 5:**
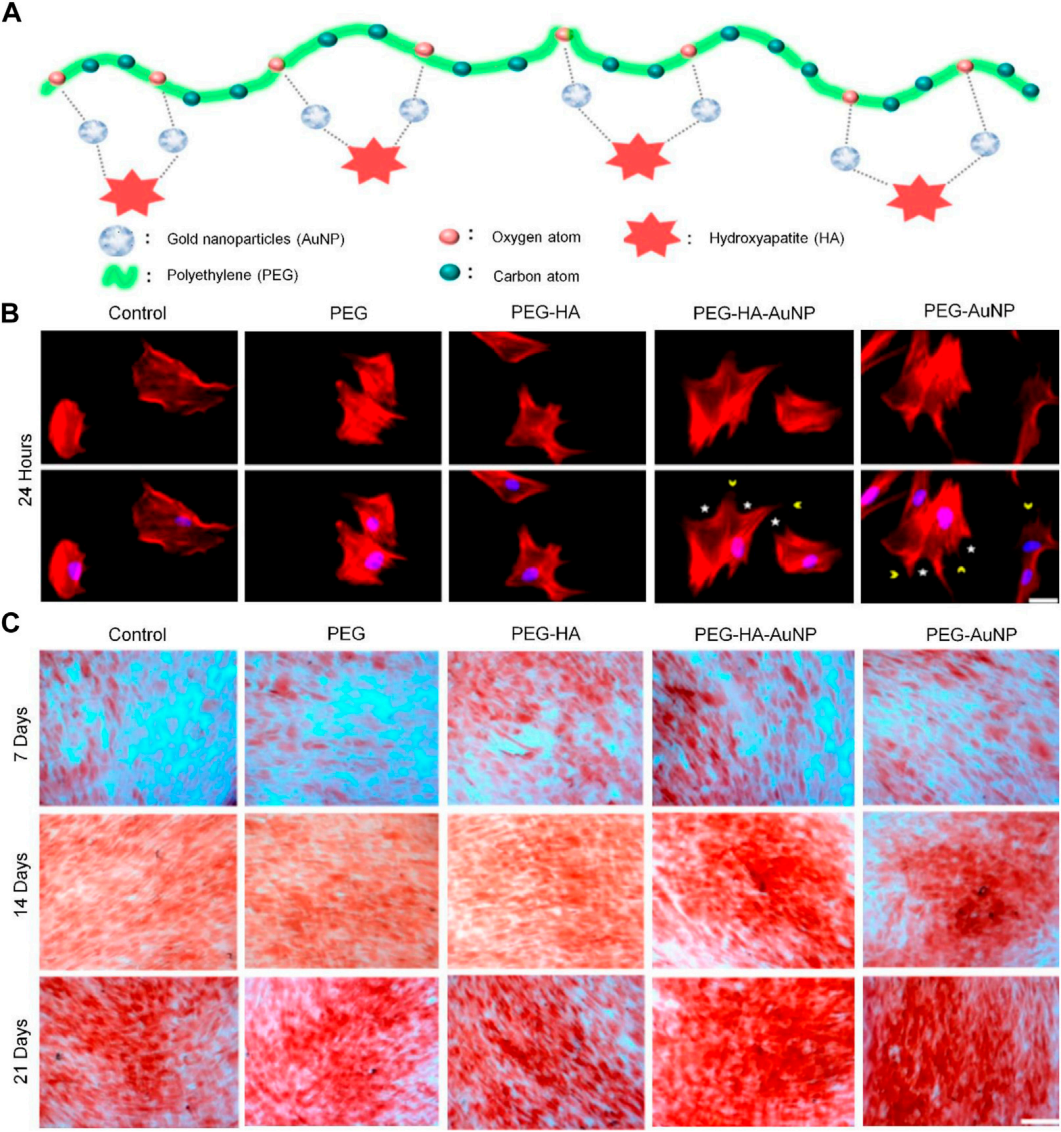
PEG-based hydrogel loaded with AuNPs for enhanced bone regeneration (Shen et al., 2021). **(A)** Schematic diagram illustrating the preparation procedure of PEG-HA-AuNPs. **(B)** Morphological changes of BMSCs after incubation on different materials for 24 h. Scale bar = 20 µm. **(C)** Osteogenic differentiation was confirmed by ARS of BMSCs on different materials after 7, 14, and 21 days of incubation. Scale bar = 20 µm. ARS, alizarin red staining; BMSCs, bone mesenchymal stem cells; HA, hydroxyapatite. Copyright ^©^ 2021 by the authors. Reproduced with permission from MDPI.

Nanocomposite biomaterials combine easily absorbed and bioactive nanofillers with biopolymers and biodegradable matrix structures (Swaminathan et al., 2020). At present, the nanomaterials that can enhance bone regeneration include nano-silica, graphene oxide, nano-titanium dioxide, nano-zirconia, and magnetic nanoparticles such as Fe_3_O_4_ (Moudgil and Ying, 2007; Gillani et al., 2010; Weitzmann et al., 2015; Elkhenany et al., 2017). Combining nanocomposites with PEG-based hydrogels can significantly promote osteogenesis. Functional nanoparticles combined with PEG-based hydrogels play an important role in the enhancement of bone regeneration. Shen et al. (2021) successfully constructed a composite hydrogel material of PEG nanogold particles (AuNPs) by surface embedding technology. *In vitro* experiments showed that the material had an induction effect on the differentiation of bone mesenchymal stem cells (BMSCs) into osteoblasts and enhanced the mineralization activity (Figure 5). Studies have shown that the size and concentration of AuNPs can significantly affect the efficiency of osteogenic differentiation. AuNPs with diameters of 18 and 45 nm combined with a PEG-based hydrogel showed excellent osteogenic ability by upregulating β-catenin and p-GSK-3β, whereas 4 nm AuNPs showed an inhibitory effect on the differentiation of BMSCs by delaying the process of bone regeneration (Zhang et al., 2021c).

As a component of ECM of bone tissue, HA plays an important role in bone regeneration. Recently, HA nanoparticles have been demonstrated to induce osteogenesis by increasing hydrophilicity, roughness, initial cell attachment, and protein adsorption (Alipour et al., 2021). The combination of a PEG-based hydrogelPEG-based hydrogel and nano-HA is a major innovative application in the field of enhancing bone regeneration. First, Ito et al. (2015) crosslinked tetraamine-terminated PEG with di-succinimide tartrate to obtain PEG hydrogel. Then, nano-HA was deposited within PEG hydrogel by heating calcium phosphate solution containing chelating agent. The addition of nano-HA with a large specific surface area into the PEG-based hydrogel network provided the required nucleation sites for the initiation of mineralization. Without affecting the original degradability and biocompatibility of the hydrogel, the nano-HA enabled the drug delivery system to have mineralization capability. The addition of mesoporous silicon dioxide nanoparticles and magnetic nanoparticles to the PEG-based hydrogel also demonstrated a strong osteogenic capability (Cao et al., 2018; Wang J. et al., 2020).

### 3.3 PEG-based hydrogels conveying nucleic acids

Genetic molecules such as microRNA and small interfering RNA (siRNA) can also be used in BTE. These non-coding RNAs can affect osteogenic differentiation by directly regulating the expression of osteogenic-related genes or by regulating the expression of inhibitors of osteogenic-related genes (Carthew et al., 2020; De La Vega et al., 2022). Therefore, loading a PEG hydrogel with nucleic acid drugs has also proved to be a promising method for the treatment of bone defects in recent years. miRNAs play crucial roles in directing mesenchymal stem cells fate, including proliferation and osteogenesis (Chen et al., 2020). Nguyen et al. (2018) synthesized a photodegradable PEG-based hydrogel through a single-crosslinking Michael addition reaction between the acrylate group in PEG-diphotolabile-acrylate and the thiol groups in eight-arm PEG-SH. The release of the genetic material was controlled by external ultraviolet irradiation. This drug delivery system induced the osteogenic differentiation of hMSCs by releasing miRNA-20a and siRNA against noggin, which is a BMP antagonist (Rai et al., 2022). miRNA-20a has been shown to enhance osteogenic differentiation of hMSCs by inhibiting the expression of peroxisome proliferator-activated receptor, Bambi, and Crim1, which negatively affects BMP signaling in osteogenesis (Liu et al., 2022). The drug delivery system could increase the release rate of RNA under ultraviolet light, but RNA continued to be released in the absence of ultraviolet light, which necessitates accurate release. To solve this problem, the properties of PEG-based hydrogels can be improved. Gan et al. (2021) formed injectable PEG-based hydrogel by the reaction of dibenzo-cyclooctyne-functionalized PEG with the azido group of eight-arm PEG. Cholesterol-modified miRNA-26a was covalently patterned onto the fibers of the PEG-based hydrogel using an ultraviolet light-cleavable linker. Therefore, Chol-miRNA-26a was selectively released by this system only under ultraviolet irradiation. Studies have shown that miRNA-26 can regulate a variety of osteogenic differentiation pathways, such as TGFβ/Smad1, Wnt, GSK3β (Zhang X. et al., 2016; Paquet et al., 2016). Cholesterol-modified miRNAs can improve the transfection efficiency of miRNA cells. Therefore, only when exposed to ultraviolet light would the system selectively release Chol-miRNA-26a. The drug delivery system showed excellent ability to enhance bone regeneration in a rat bone defect model.

siRNA can both induce specific silencing of a wide range of genetic targets and play a role in promoting bone regeneration by inhibiting osteogenic inhibitory factors (Xing et al., 2020). Therefore, siRNA is delivered to the bone defect by a PEG-based hydrogel carrier, which acts on local stem cells, promotes the expression of osteogenic-related genes, and promotes bone regeneration (Manaka et al., 2011). Wang et al. (2017) first combined siRNA with polymer nanoparticles by electrostatic interaction, and then embedded them in a PEG-PLA-DM hydrogel. The drug delivery system was implanted into a femoral defect in rats, and the results showed that the sustained release lasted for 28 days. Compared with the control group, the siRNA/hydrogel group showed significant biomechanical strength and rapid bone regeneration.

### 3.4 PEG-based hydrogels for delivery of osteogenic drugs

Compared with growth factors and nanoparticles, osteogenic drugs (with low costs and stable release *in vivo*) have their own unique advantages in enhancing bone regeneration (Cisneros et al., 2021). Therefore, a PEG-based hydrogel loaded with osteogenic drugs could also promote osteogenesis at the local bone defects. Simvastatin, an inhibitor of 3-hydroxy-methylglutaryl-CoA reductase, is often used clinically to treat hypercholesterolemia and has recently been shown to have excellent metabolic promoting effects on bones (Wang C.-Z. et al., 2018). In one study, simvastatin was dissolved in acetone and added to the PLGA-PEG-PLGA hydrogel solution by vortexing. The hydrophobic micelle core of PLGA provides a microenvironment for simvastatin incorporation. The PEG-based hydrogel drug delivery system could promote defect repair by continuously releasing simvastatin in the defect area (Yan et al., 2015).

**FIGURE 6:**
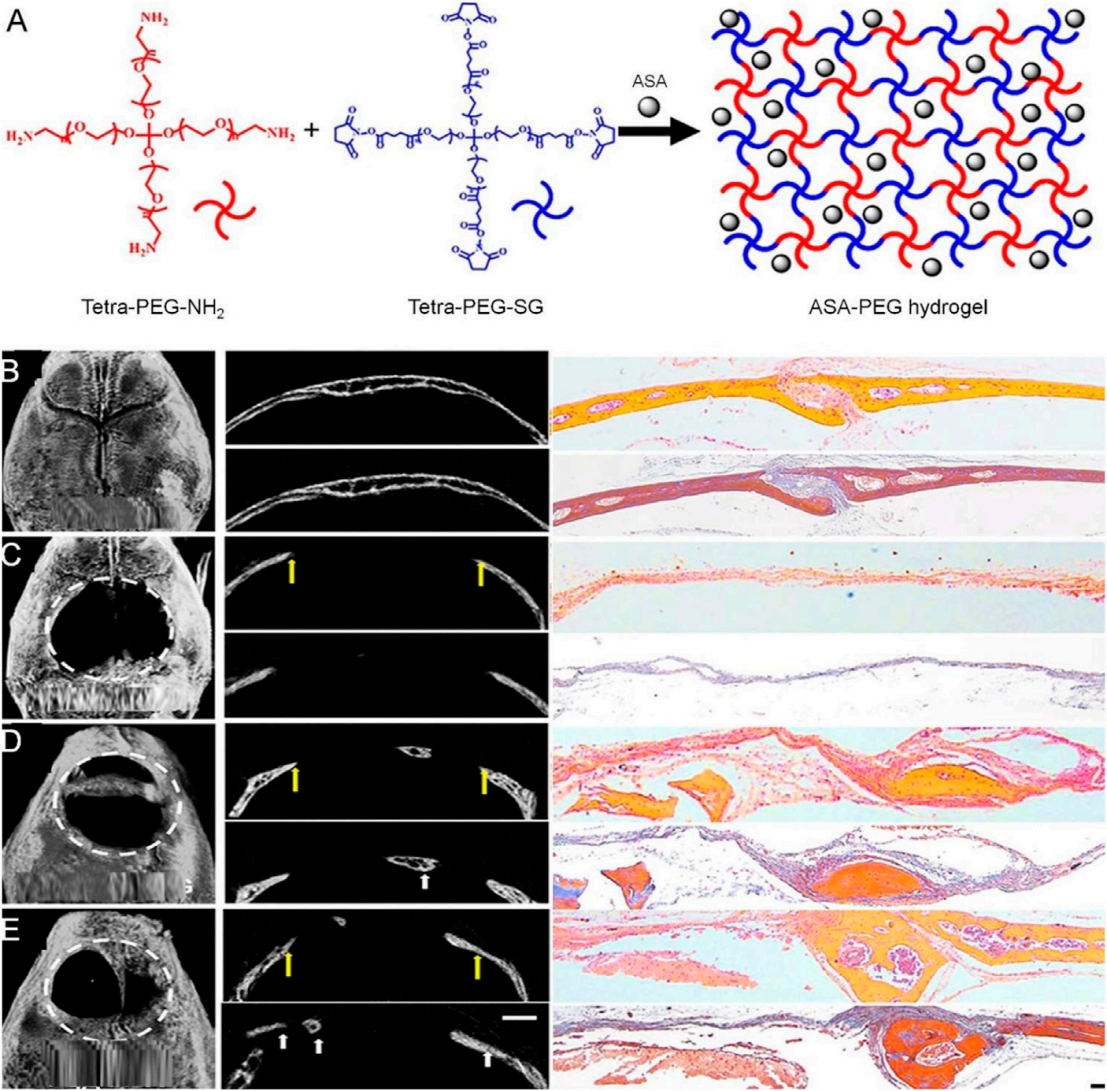
Tetra-PEG hydrogel-based aspirin sustained release system with beneficial effects on bone regeneration (Zhang et al., 2019). **(A)** Scheme of the synthesis procedure for ASA-PEG hydrogels. **(B)** μCT (left), H&E (upper), and Masson staining (lower) images of mice calvaria bone structure, **(C)** untreated experimentally induced calvaria bone defects, **(D)** calvaria bone defects after transplantation of PDLSCs and PEG hydrogels, and **(E)** calvaria bone defects after transplantation of PDLSCs and PEG-ASA hydrogels. The yellow arrows in **(C–E)** indicate the margins of bone defects, and the white arrows indicate new bone. The white dotted line defines the bone defect area. ASA, acetylsalicylic acid; PDLSCs, periodontal ligament stem cells; H&E, hematoxylin and eosin; μCT, micro-computed tomography. Copyright ^©^ 2019 Zhang, Ding, Zhang, Sun, Han, and Yu. Reproduced with permission from FRONTIERS MEDIA SA.

Aspirin (ASP) has long been clinically used as a non-steroidal anti-inflammatory drug. However, it has been reported that ASP can treat osteoporosis in animals by affecting the activities of osteoblasts and osteoclasts (Lei et al., 2019). Zhang et al. (2019) developed a PEG-ASP hydrogel with local sustained release by directly dissolving ASP in tetra-PEG-succinimimidyl glutarate. The experimental results showed that the drug delivery system could significantly enhance the osteogenic differentiation of mesenchymal cells and slightly promote cell proliferation. The PEG-ASP sustained-release system was implanted into skull defects in mice. 8 weeks later, micro-CT showed that the new bone formation area in the experimental group was significantly higher than that in the control group (Figure 6). Thus, the PEG-ASP sustained-release system provides a new strategy for bone regeneration.

Bisphosphonates have significant therapeutic effects on primary or secondary osteoporosis (Chu et al., 2017). The long-term, local administration of a minimally invasive gel system can continuously transport alendronate (ALN) to targeted sites to enhance bone regeneration. In one study, PEG-ALN was formed by an amidation reaction between four-arm PEG and ALN (Li D. et al., 2020). PEG could improve the release rate and hydrophilicity of ALN. *In vitro* experiments showed that the composite system released ALN for 28 days, and PEG-ALN could enhance bone regeneration limitedly by implanting a PEG-based hydrogel into an ovariectomized rabbit model. Similarly, building a delivery system composed of a PEG-based hydrogel with calcitriol and other osteogenic drugs can also demonstrate a strong ability to promote osteogenesis (Hu et al., 2021).

### 3.5 PEG-based hydrogels for delivery of anti-infective drugs

**FIGURE 7:**
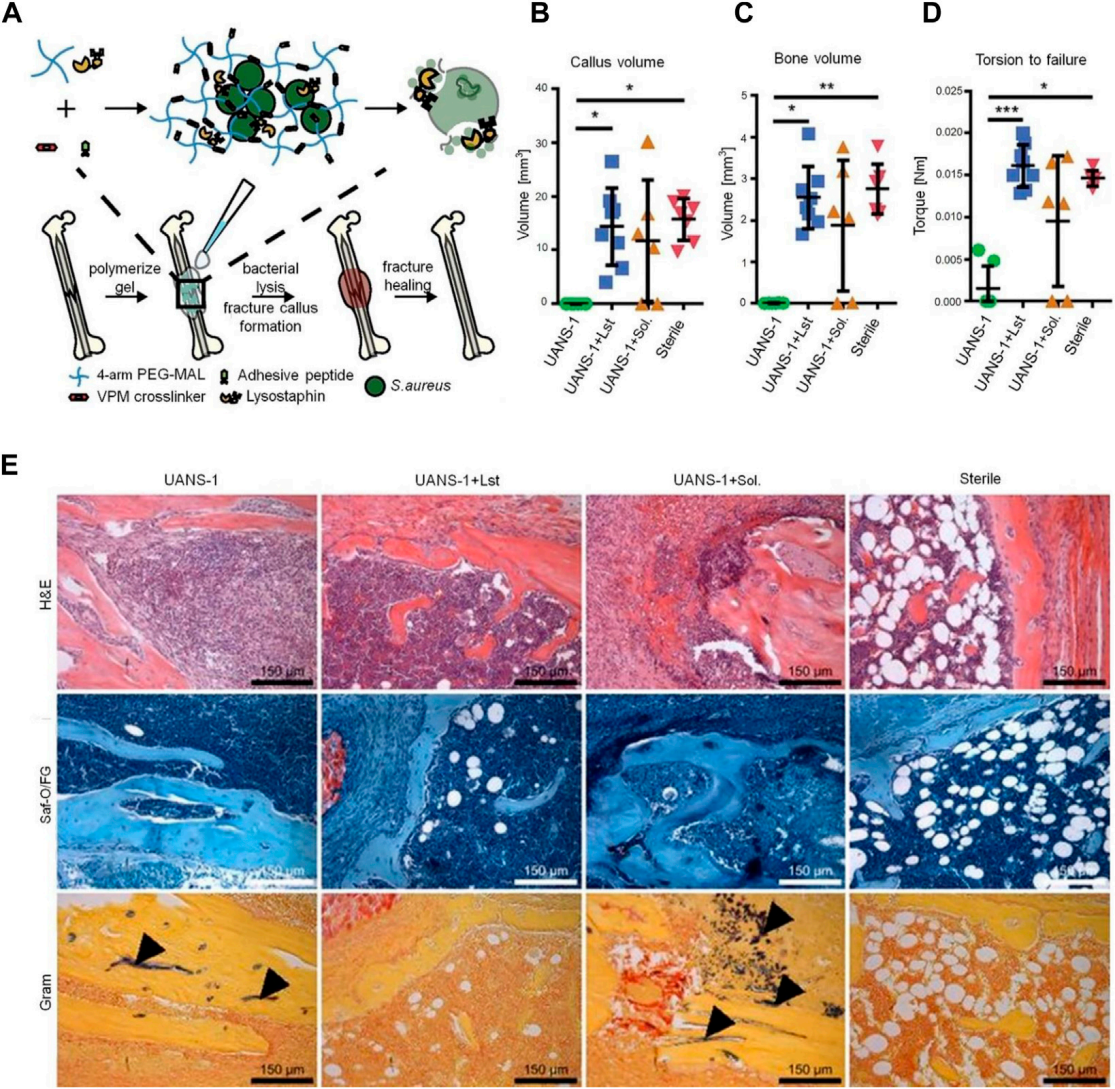
PEG-based hydrogel delivery of lysostaphin eliminates orthopedic implant infection by *S. aureus* and supports fracture healing (Johnson et al., 2018). **(A)** Schematic diagram of lysostaphin encapsulation within protease-degradable PEG-MAL hydrogel and subsequent application to infected femurs. Quantification of μCT reconstructions showing the **(B)** fracture callus volume and **(C)** bone volume within the fracture callus at 5 weeks. **(D)** Experimental evaluation of mechanical strength of the femur. *p < 0.05, **p < 0.01, ***p < 0.001. **(E)** H&E, Saf-O/FG, and Gram staining of femurs. Black arrows indicate Gram-positive bacteria. MAL, maleimide; H&E, hematoxylin and eosin; Saf-O/FG, safranin O/fast green. Copyright ^©^ 2018 the Author(s). Reproduced with permission from PNAS.

Infection is one of the most important reasons for bone non-union (Jairo Aguilera-Correa et al., 2020). It is reported that the infection rate of open fractures varies from 20% to 50% (Ahadi et al., 2019). The most important link in the treatment of infectious bone defects is to control infection. The systemic application of antibiotics makes it difficult to achieve an effective local concentration and increases antibiotic resistance. Antibiotic-loaded bone cement has an obvious therapeutic effect *in vivo*, but bone cement is non-degradable, which will necessitate secondary surgery (Wang et al., 2016). In comparison, PEG-based hydrogels have good biocompatibility and do not cause inflammatory reactions *in vivo*. PEG-based hydrogel drug delivery systems are promising for the control of bone defects accompanied by infection. Vancomycin, an amphoteric glycopeptide antibiotic, has a strong killing effect on Gram-positive bacteria, especially on methicillin-resistant *S. aureus* (Li Y. et al., 2020). Li et al. (2017) coated a vancomycin-loaded PEG-based hydrogel onto titanium implants. An *in vitro* drug release test showed that vancomycin sustained release for nearly 3 weeks without an initial explosive release. The vancomycin-loaded titanium graft was implanted into a rabbit model with *S. aureus* infection. The white blood cell count in the experimental group returned to its preoperative level 4 weeks after operation. Compared with vancomycin, lysostaphin, a metallopeptidase produced by mimic *Staphylococcus*, has a wider antimicrobial spectrum (Johnson et al., 2019); it can kill stationary bacteria, planktonic bacteria, and bacteria growing in the biofilm, whereas, most antibiotics are effective against metabolically active bacteria. Johnson et al. (2018) physically encapsulated lysostaphin into hydrogel crosslinked by PEG, cell adhesion peptides and protease-degradable peptides. The drug delivery system could maintain lysostaphin activity for more than 14 days. In the infection model, the experimental group restored the local infection environment to the physiological environment of aseptic fracture after 7 days. Complete healing occurred within 5 weeks, and the bone formation and mechanical properties were similar to those of a pure fracture (Figure 7).

Bacteriophages can also be used as an alternative to traditional antibiotics. Bacteriophages are host-specific, indicating that they only infect specific bacteria, which avoids negative effects on the human microbiota (Cobb et al., 2019). *Pseudomonas aeruginosa* (*P. aeruginosa*) is reported to be a common Gram-negative bacterium of orthopedic infections, with a recurrence probability that is 2.5 times that of *S. aureus* infections (Ferry et al., 2022). To better treat bone defects infected by *P. aeruginosa*, the targeted active bacteriophage can be wrapped in an injectable PEG-based hydrogel and transported to the site of bone infection. In a mouse infection model, it was found that, compared with the control group, the infection site of *P. aeruginosa* in the experimental group was reduced by 4.7 times after 7 days of implantation (Wroe et al., 2020). The phage delivery system provides a new strategy for the treatment of local bone infections.

## 4 Conclusion and perspectives

Maintaining an appropriate concentration of osteogenically active material at the defect site is critical for bone regeneration. Hydrogels are considered one of the best options for constructing drug delivery systems due to their good plasticity, appropriate loose network structure, and degradability. PEG has good hydrophilicity and many terminal groups. By mixing with other materials, the PEG hydrogel can obtain an improved carrier performance. In addition, PEG-based hydrogels can also have antibacterial and bone-promoting properties by crosslinking or binding with antibacterial and osteogenic substances. PEG-based hydrogels can be rapidly gelled *in situ* after implantation, which can reduce the leakage of the load material and avoid the initial burst effect of drug loading. At the same time, PEG-based hydrogels have excellent biocompatibility and can be degraded after achieving the desired effect, where the degradation products are non-cytotoxic. PEG-based hydrogels can be used to construct drug delivery systems with a variety of substances, including osteogenic factors and derivatives, nucleic acids, osteogenic drugs and antibacterial drugs, to enhance bone regeneration. Although, in recent years, poly (2-oxazoline)s and poly (2-oxazine)s have been considered as alternative polymers to PEG. However, PEG can disguise drug delivery systems by eliminating protein-like reactions with the immune system, which is very beneficial for drug delivery systems. A variety of studies have shown that the PEG-based drug delivery system does not cause an inflammatory reaction in the body, and the drug release curve conforms to the framework time of bone regeneration, thus playing an indispensable role in the growth of new bones.

Although significant progress has been made in recent years in the study of PEG-based hydrogel drug delivery systems for the local enhancement of bone regeneration, most of them are at the basic research stage of animal experiments, and there is still a lack of large-scale clinical studies to prove the efficacy and safety of PEG-based hydrogels. Many issues still need to be considered for the introduction of PEG-based hydrogels into medical treatments. For example, the biomedical sector must address issues such as technology transfer and process amplification. At the same time, the law is also one of the important factors that we must consider. These will be the direction of future research. In addition, the drug release curves of PEG-based hydrogels have been mostly verified *in vitro*, and the *in vivo* release of drugs can be explored by fluorescence labeling in future studies. Subsequent studies should also focus on improving the physical and chemical properties of PEG-based hydrogels, so as to achieve more intelligent drug delivery systems for bone repair. We believe that PEG-based hydrogel drug delivery systems will provide new ways to promote the field of bone regeneration.
